# Factors associated with the consumption of chlorine dioxide to prevent and treat COVID-19 in the Peruvian population: a cross-sectional study

**DOI:** 10.1186/s12889-021-12191-9

**Published:** 2021-11-17

**Authors:** David R. Soriano-Moreno, Daniel Fernandez-Guzman, Fabricio Ccami-Bernal, Cristhian Rojas-Miliano, Wendy Nieto-Gutierrez

**Affiliations:** 1 Unidad de Investigación Clínica y Epidemiológica, Escuela de Medicina, Universidad Peruana Unión, Lima, Peru; 2 Grupo Peruano de Investigación Epidemiológica, Unidad para la Generación y Síntesis de Evidencias en Salud, Universidad San Ignacio de Loyola, Lima, Peru; 3 Universidad Nacional de San Agustín de Arequipa, Arequipa, Peru; 4 Universidad Nacional del Centro del Peru, Huancayo, Peru; 5 Universidad San Ignacio de Loyola, Unidad de Investigación para la Generación y Síntesis de Evidencias en Salud, Av. la Fontana 550, La Molina, Lima, Peru

**Keywords:** COVID-19, SARS-CoV-2, Pandemic, Associated factors, Chlorine dioxide (source: MESH)

## Abstract

**Background:**

Chlorine dioxide has been promoted as an alternative for the prevention and treatment of COVID-19, especially in Peru, despite the lack of evidence to support its efficacy. This study aimed to evaluate the factors associated with chlorine dioxide consumption in the Peruvian population.

**Methods:**

Analytical cross-sectional study. An adult Peruvian population was evaluated where chlorine dioxide consumption was divided into two groups according to the purpose of use: as prevention (individuals without COVID-19 history) and as treatment (individuals with COVID-19 history). The associated factors in each group were evaluated using Poisson regressions with the bootstrapping resampling method.

**Results:**

Of 3610 participants included, 3213 reported no history of COVID-19, and 397 had been infected. The prevalence of chlorine dioxide consumption to prevent or treat COVID-19 was 8 and 16%, respectively. Factors either positively or negatively associated with chlorine dioxide consumption for prevention were male sex (aPR: 1.36; 95% CI: 1.09–1.71), being an adult or older adult (aPR: 0.54; 95% CI: 0.35–0.82), having a health sciences student within the family unit (aPR: 1.38; 95% CI: 1.02–1.87), using medical information as the main source of information of COVID-19 (aPR: 0.57; 95% CI: 0.40–0.80), having comorbidities for COVID-19 (aPR: 1.36; 95% CI: 1.01–1.82), considering COVID-19 dangerous and deadly (aPR: 0.57; 95% CI: 0.45–0.74), using medications (aPR: 1.59; 95% CI: 1.25–2.06) and plants to prevent COVID-19 (aPR: 1.69; 95% CI: 1.21–2.36), considering chlorine dioxide ineffective (aPR: 0.18; 95% CI: 0.18–0.24), and being uninformed of its efficacy (aPR: 0.21; 95% CI: 0.16–0.28). In addition, factors associated with chlorine dioxide consumption for treatment were considering COVID-19 dangerous and deadly (aPR: 0.56; 95% CI: 0.33–0.96), considering chlorine dioxide ineffective (aPR: 0.22; 95% CI: 0.12–0.42), and being uninformed of its efficacy (aPR: 0.15; 95% CI: 0.07–0.32).

**Conclusions:**

The prevalence of chlorine dioxide consumption to treat COVID-19 was higher than prevent. It is important to apply information strategies, prioritizing population groups with certain characteristics that are associated with a higher consumption pattern.

**Supplementary Information:**

The online version contains supplementary material available at 10.1186/s12889-021-12191-9.

## Introduction

The COVID-19 pandemic has caused millions of deaths around the world, with the Americas being the region with the highest mortality rate [1], which has generated anguish and fear in the population. This, added to the atmosphere of uncertainty and the dissemination of a large amount of false information [2], has led the population to seek and adopt remedies that promise to be wildly effective to avoid contagion or death due to COVID-19 [3], leading to the use of substances such as chlorine dioxide [2].

Chlorine dioxide is a chemical compound that occurs as a highly reactive gas, so it is generally marketed and used in its liquid form or solution known as CDS (chlorine dioxide solution) [4]. Due to its strong oxidizing power, chlorine dioxide and its derivatives are used as disinfectant agents in different industrial processes [5]; however, during the pandemic, it has been promoted as an alternative for the prevention and treatment of COVID-19, whose use has been reported by inhalation, oral and parenteral application [6]. This substance does not have sanitary approval for the prevention or treatment of any disease [7] and its efficacy has not been proven, while its risk of toxicity has been proven [8].

Peru is a country characterized by social and health inequalities [9], which have repercussions on access to health services in terms of policies, distribution of human resources, and infrastructure [10]. This, added to the widespread misinformation and promotion of pseudo-scientific therapies during the pandemic, resulted in an increased risk of ineffective and dangerous measures against COVID-19 that imply a risk to the health of citizens [2]. Even in 2021, the effects of the use of chlorine dioxide related to COVID-19 in the country have continued to be evaluated [11]. The present study aims to evaluate the factors associated with the consumption of chlorine dioxide in the Peruvian population, to foster strategies focused on the most prevalent groups, with the purpose of avoiding harmful outcomes in the population.

## Methodology

### Study design, space, and participants

An analytical observational study was carried out from a previously collected database, obtained to describe the prevention and control practices against COVID-19 in the Peruvian population. This study was conducted in the general adult population (> 18 years) of Peruvian residence and nationality in the 24 departments of the country, excluding women who reported pregnancy at the time of the survey. The survey was distributed virtually over a two-week period (September 7–21, 2020) using non-probability snowball sampling. More details about the methodology have been detailed previously [12].

### Questionnaire

The questionnaire used was designed and validated by the authors and was divided into three sections: 1) general data (biological, demographic, epidemiological characteristics), 2) prevention perspectives and practices of participants without a history of COVID-19, and 3) perspectives and practices during COVID-19 infection of participants with a history of COVID-19. The first section of the questionnaire was answered by all respondents, the second section was answered only by respondents without a history of COVID-19 and the third section was answered only by respondents with a history of COVID-19 (Supplementary Material 1).

### Dependent variable: chlorine dioxide consumption

The dependent variable was assessed in two populations: individuals without a history of COVID-19 and those with a history of COVID-19. For those without a history of COVID-19, chlorine dioxide consumption was assessed as COVID-19 prevention with the question “*In the last 2 weeks on how many occasions have you used chlorine dioxide for COVID-19 prevention?*”. If the respondent answered having used it at least once, the respondent was considered to have used chlorine dioxide for prevention. For those with a history of COVID-19, chlorine dioxide consumption was assessed as a treatment of COVID-19 with the question “*During your COVID-19 infection, on how many occasions have you used chlorine dioxide against COVID-19?*”. Chlorine dioxide was considered to have been used as a treatment if the respondent answered that it was used at least once.

### Statistical analysis

The Stata v16.0 statistical package was used for data analysis. In the descriptive analysis, absolute and relative frequencies were determined. In the bivariate analysis, the chi-square test was used to compare the proportions between groups. For the evaluation of factors associated with chlorine dioxide consumption, Poisson regression with robust variances using the bootstrapping resampling method was used to obtain prevalence ratios (PR) with their respective 95% confidence intervals (95%CI).

### Ethical aspects

The primary study was evaluated and approved by the institutional ethics committee of the Universidad Peruana Unión (Certificate of Approval: 2020-CEUPeU-00020). All methods were performed in accordance with the Declaration of Helsinki. Informed consent was obtained from the participants for data collection.

## Results

The database used for the present study had a total of 3630 individuals (3231 without COVID-19 and 399 with COVID-19); however, 20 women were excluded because they reported being pregnant. Finally, 3610 individuals were evaluated, of whom 3213 (89%) reported not having had a history of COVID-19 and 397 (11%) reported having had a history of COVID-19 (Supplementary Material 2).

In the population evaluated, the majority were female (62.4 and 58.9%), young people aged 18 to 29 years (84.7 and 72.8%), with higher education (76.1 and 77.6%), from the coastal region (48.4 and 46.1%), from urban areas (84.8 and 83.1%), and of upper-middle social class (73.6 and 76.1%), both for those who did not have a history of COVID-19 and those who did, respectively (Table 1).

**Table 1:** Characteristics of participants according to consumption of chlorine dioxide for prevention and as a treatment against COVID-19 in the Peruvian population (*N* = 3610)

Variables	Participants with no history of COVID-19 (***N*** = 3213)				Participants with a history of COVID-19 (***N*** = 397)			
	N (%)	Use of chlorine dioxide for COVID-19 prevention			N (%)	Use of chlorine dioxide as a treatment for COVID-19		
		No use	Use	***P***^**a**^		No use	Use	***P***^**a**^
		2956 (92%) n (%)	257 (8%) n (%)			332 (83.6%) n (%)	65 (16.4%) n (%)	
Sex								
Female	2009 (62.53)	1870 (93.08)	139 (6.92)	**0.004**	234 (58.94)	201 (85.90)	33 (14.10)	0.143
Male	1204 (37.47)	1086 (90.20)	118 (9.80)		163 (41.06)	131 (80.37)	32 (19.63)	
Age								
Young (18 to 29 years old)	2720 (84.66)	2494 (91.69)	226 (8.31)	0.128	289 (72.80)	251 (86.85)	38 (13.15)	**0.005**
Adult and older adult (> 29 years)	493 (15.34)	462 (93.71)	31 (6.29)		108 (27.20)	81 (75.00)	27 (25.00)	
Marital status								
Single	2829 (88.05)	2601 (91.94)	228 (8.06)	0.731	303 (76.32)	256 (84.49)	47 (15.51)	0.405
Married or cohabiting	384 (11.95)	355 (92.45)	29 (7.55)		94 (23.68)	76 (80.85)	18 (19.15)	
Education level								
High school or less	769 (23.93)	709 (92.20)	60 (7.80)	0.818	89 (22.42)	75 (84.27)	14 (15.73)	0.852
Superior	2444 (76.07)	2247 (91.94)	197 (8.06)		308 (77.58)	257 (83.44)	51 (16.56)	
Employment status								
Not working	2502 (77.87)	2308 (92.25)	194 (7.75)	0.337	240 (60.45)	212 (88.33)	28 (11.67)	**0.002**
Working	711 (22.13)	648 (91.14)	63 (8.86)		157 (39.55)	120 (76.43)	37 (23.57)	
Health insurance								
None	972 (30.25)	889 (91.46)	83 (8.54)	0.323	100 (25.19)	88 (88.00)	12 (12.00)	0.587
SIS	1199 (37.32)	1100 (91.74)	99 (8.26)		131 (33.00)	107 (81.68)	24 (18.32)	
EsSalud	637 (19.83)	585 (91.84)	52 (8.16)		101 (25.44)	83 (82.18)	18 (17.82)	
Others	405 (12.61)	382 (94.32)	23 (5.68)		65 (16.37)	54 (83.08)	11 (16.92)	
Region of residence								
Coast	1572 (48.93)	1477 (93.96)	95 (6.04)	**< 0.001**	183 (46.10)	160 (87.43)	23 (12.57)	**0.008**
Highland	1363 (42.42)	1230 (90.24)	133 (9.76)		135 (34.01)	115 (85.19)	20 (14.81)	
Jungle	278 (8.65)	249 (89.57)	29 (10.43)		79 (19.90)	57 (72.15)	22 (27.85)	
Area of residence								
Urban	2536 (84.81)	2536 (93.06)	189 (6.94)	**< 0.001**	330 (83.12)	281 (85.15)	49 (14.85)	0.069
Rural	488 (15.19)	420 (86.07)	68 (13.93)		67 (16.88)	51 (76.12)	16 (23.88)	
Social class								
Medium-High	2364 (73.58)	2192 (92.72)	172 (7.28)	**0.012**	302 (76.07)	253 (83.77)	49 (16.23)	0.887
Lower	849 (26.42)	764 (89.99)	85 (10.01)		95 (23.93)	79 (83.16)	16 (16.84)	
Health professional within the family unit								
No	1250 (38.90)	1123 (89.84)	127 (10.16)	**< 0.001**	138 (34.76)	107 (77.54)	31 (22.46)	**0.036**
Student of health sciences	513 (15.97)	462 (90.06)	51 (9.94)		79 (19.90)	66 (83.54)	13 (16.46)	
Health professional	1450 (45.13)	1371 (94.55)	79 (5.45)		180 (45.34)	159 (88.33)	21 (11.67)	
Source where you obtain information about COVID-19 medication								
Other media	2368 (73.70)	2139 (90.33)	229 (9.67)	**< 0.001**	288 (72.54)	230 (79.86)	58 (20.14)	**0.001**
Medical information	845 (26.30)	817 (96.69)	28 (3.31)		109 (27.46)	102 (93.58)	7 (6.42)	
Comorbidities for COVID-19								
No	2743 (85.37)	2531 (92.27)	212 (7.73)	0.173	306 (77.08)	267 (87.25)	39 (12.75)	**< 0.001**
Yes	470 (14.63)	425 (90.43)	45 (9.57)		91 (22.92)	65 (71.43)	26 (28.57)	
Family member with comorbidity for COVID-19								
No	1574 (48.99)	1413 (89.77)	161 (10.23)	**< 0.001**	181 (45.59)	152 (83.98)	29 (16.02)	0.863
Yes	1639 (51.01)	1543 (94.14)	96 (5.86)		216 (54.41)	180 (83.33)	36 (16.67)	
Family member with COVID-19 diagnosis								
No	2385 (74.23)	2204 (92.41)	181 (7.59)	0.146	40 (10.08)	33 (82.50)	7 (17.50)	0.839
Yes	828 (25.77)	752 (90.82)	76 (9.18)		357 (89.92)	299 (83.75)	58 (16.25)	
Family member deceased by COVID-19								
No	3171 (98.69)	2917 (91.99)	254 (8.01)	0.837	371 (93.45)	313 (84.37)	58 (15.63)	0.133
Yes	42 (1.31)	39 (92.86)	3 (7.14)		26 (6.55)	19 (73.08)	7 (26.92)	
Do you consider COVID-19 to be a dangerous and deadly disease?								
Nothing or little	748 (23.28)	653 (87.30)	95 (12.70)	**< 0.001**	97 (24.43)	75 (77.32)	22 (22.68)	0.053
Much	2465 (76.72)	2303 (93.43)	162 (6.570)		300 (75.57)	257 (85.67)	43 (14.33)	
Use of medications for prevention or treatment of COVID-19								
No	2501 (77.84)	2347 (93.84)	154 (6.16)	**< 0.001**	64 (16.12)	57 (89.06)	7 (10.94)	0.199
Yes	712 (22.16)	609 (85.53)	103 (14.47)		333 (83.88)	275 (82.58)	58 (17.42)	
Use of medicinal plants for prevention or treatment of COVID-19								
No	1294 (40.27)	1244 (96.14)	50 (3.86)	**< 0.001**	79 (19.90)	70 (88.61)	9 (11.39)	0.181
Yes	1919 (59.73)	1712 (89.21)	207 (10.79)		318 (80.10)	262 (82.39)	56 (17.61)	
Opinion of chlorine dioxide as prevention or treatment of COVID-19								
It is effective	396 (12.32)	271 (68.43)	125 (31.57)	**< 0.001**	58 (14.61)	22 (37.93)	36 (62.07)	**< 0.001**
It is not effective	1681 (52.32)	1614 (96.01)	67 (3.99)		182 (45.84)	164 (90.11)	18 (9.89)	
I am not informed of the subject	1136 (35.36)	1071 (94.28)	65 (5.72)		157 (39.55)	146 (92.99)	11 (7.01)	
COVID-19 disease severity								
Mild	Not applicable				372 (93.70)	320 (86.02)	52 (13.98)	**< 0.001**
Moderate or severe					25 (6.30)	12 (48.00)	13 (52.00)	

^a^Calculated with the chi^2^ statistic; significant *p*-value in bold (*P* < 0.05)

The prevalence of chlorine dioxide consumption as a COVID-19 preventive measure was 8%, with a higher prevalence of consumption observed in male individuals (9.8%, *P* = 0.004), from the highlands or jungle region (9.8, 10.4%, *P* < 0.001), rural area of residence (13.9%, *P* < 0.001), low social class (10.1%, *P* = 0.012), without a health professional in the family unit (10.2%, *P* < 0.001), who did not have medical information as their main source of information about COVID-19 (9.6%, *P* < 0.001), without a family member with comorbidities for COVID-19 (10.2%, *P* < 0.001), who considered COVID-19 as a not at all or only slightly dangerous and deadly disease (12.7%, *P* < 0. 001), who used medications as prevention for COVID-19 (14.5%, *P* < 0.001), who used medicinal plants as prevention for COVID-19 (10.8%, *P* < 0.001), and who thought that chlorine dioxide is effective (31.6%, *P* < 0.001) (Table 1).

Likewise, the prevalence of chlorine dioxide consumption as a treatment was 16.4%, and the percentage was higher in adult individuals aged 30 years and older (25.0%, *P* = 0.005), workers (23.6%, *P* = 0.017), from the jungle region (27.9%, *P* < 0.001), without a health professional in the family unit (22.5%, *P* = 0. 036), who did not have medical information as their main source of information about COVID-19 (20.1%, *P* = 0.001), with comorbidities for COVID-19 (28.6%, *P* < 0.001), who thought that chlorine dioxide is effective (62.1%, *P* < 0.001) and who thought that their case of COVID-19 was moderate-severe (52.0%, *P* < 0.001) (Table 1).

When evaluating the factors associated with chlorine dioxide consumption as a preventive measure, we found a statistically significant association (either positive or negative) with male sex (aPR: 1.36; 95%CI: 1.09–1.71), being an adult or older adult (over 30 years of age) (aPR: 0.54; 95%CI: 0.35–0.82), having a health sciences student within the family unit (aPR: 1.38; 95%CI: 1.02–1.87), having medical information as a source of information about COVID-19 (aPR: 0.57; 95%CI: 0.40–0.80), having comorbidities for COVID-19 (aPR: 1.36; 95%CI: 1.01–1.82), strongly considering COVID-19 to be a dangerous and deadly disease (aPR: 0.57; 95%CI: 0.45–0.74), using medications to prevent COVID-19 (aPR: 1.59; 95%CI: 1.25–2.06), using plants to prevent COVID-19 (aPR: 1.69; 95%CI: 1.21–2.36), thinking that chlorine dioxide is not effective (aPR: 0.18; 95%CI: 0.18–0.24), and not being informed of the efficacy of chlorine dioxide (aPR: 0.21; 95%CI: 0.16–0.28) (Table 2).

**Table 2:** Factors associated with the consumption of chlorine dioxide for prevention and treatment against COVID-19 in the Peruvian population (*N* = 3610)

Variables	Chlorine dioxide consumption			
	For prevention		For treatment	
	Bivariate analysis	Multivariate analysis	Bivariate analysis	Multivariate analysis
	cPR (95% CI)	aPR (95% CI)	cPR (95% CI)	aPR (95% CI)
Sex				
Female	Ref.	Ref.	Ref.	Ref.
Male	1.42 (1.12–1.79)	**1.36 (1.09–1.71)**	1.39 (0.92–2.10)	1.21 (0.77–1.89)
Age				
Young (18 to 29 years old)	Ref.	Ref.	Ref.	Ref.
Adult and older adult (> 29 years)	0.76 (0.52–1.10)	**0.54 (0.35–0.82)**	1.90 (1.20–3.02)	0.88 (0.46–1.69)
Health insurance				
None	Ref.	Ref.	Ref.	Ref.
SIS	0.97 (0.71–1.31)	0.85 (0.63–1.16)	1.53 (0.78–2.98)	1.26 (0.58–0.58)
EsSalud	0.96 (0.67–1.37)	1.08 (0.75–1.57)	1.49 (0.74–2.97)	1.47 (0.68–3.18)
Others	0.67 (0.43–1.03)	0.85 (0.56–1.30)	1.41 (0.59–3.36)	1.10 (0.52–2.33)
Region of residence				
Coast	Ref.	Ref.	Ref.	Ref.
Highland	1.61 (1.24–2.10)	1.23 (0.94–1.60)	1.18 (0.66–2.11)	1.14 (0.61–2.12)
Jungle	1.73 (1.15–2.59)	1.07 (0.73–1.56)	2.22 (1.28–3.83)	1.33 (0.74–2.37)
Area of residence				
Urban	Ref.	Ref.	Ref.	Ref.
Rural	2.01 (1.53–2.63)	1.22 (0.93–1.61)	1.61 (0.97–2.66)	1.1 (0.54–2.21)
Social class				
Medium-High	Ref.	Ref.	Ref.	Ref.
Lower	1.38 (1.08–1.75)	0.96 (0.74–1.24)	1.04 (0.63–1.70)	0.75 (0.41–1.35)
Health professional within the family unit				
No	Ref.	Ref.	Ref.	Ref.
Student of health sciences	0.98 (0.72–1.32)	**1.38 (1.02–1.87)**	0.73 (0.38–1.41)	0.8 (0.41–1.56)
Health professional	0.54 (0.40–0.72)	0.79 (0.58–1.06)	0.52 (0.31–0.87)	0.64 (0.39–1.06)
Source where you obtain information about COVID-19 medication				
Other media	Ref.	Ref.	Ref.	Ref.
Medical information	0.34 (0.24–0.48)	**0.57 (0.40–0.80)**	0.32 (0.03–3.07)	0.48 (0.05–4.36)
Comorbidities for COVID-19				
No	Ref.	Ref.	Ref.	Ref.
Yes	1.24 (0.94–1.64)	**1.36 (1.01–1.82)**	2.24 (1.46–3.44)	1.36 (0.79–2.35)
Do you consider COVID-19 to be a dangerous and deadly disease?				
Nothing or little	Ref.	Ref.	Ref.	Ref.
Much	0.52 (0.41–0.66)	**0.57 (0.45–0.74)**	0.63 (0.41–0.98)	**0.56 (0.33–0.96)**
Use of medications for prevention or treatment of COVID-19				
No	Ref.	Ref.	Ref.	Ref.
Yes	2.35 (1.83–3.02)	**1.59 (1.23–2.06)**	1.59 (0.75–3.39)	2.04 (0.90–4.62)
Use of medicinal plants for prevention or treatment of COVID-19				
No	Ref.	Ref.	Ref.	Ref.
Yes	2.79 (2.05–3.81)	**1.69 (1.21–2.36)**	1.55 (0.76–3.13)	1.03 (0.52–2.04)
Opinion of chlorine dioxide as prevention or treatment of COVID-19				
It is effective	Ref.	Ref.	Ref.	Ref.
It is not effective	0.13 (0.10–0.17)	**0.18 (0.13–0.24)**	0.16 (0.10–0.26)	**0.22 (0.12–0.42)**
I am not informed of the subject	0.18 (0.14–0.24)	**0.21 (0.16–0.28)**	0.11 (0.06–0.22)	**0.15 (0.07–0.32)**
COVID-19 disease severity				
Mild	Not applicable		Ref.	Ref.
Moderate or severe			3.72 (2.24–6.17)	1.45 (0.69–3.04)

*cPR* Crude prevalence ratio, *aPR* Adjusted prevalence ratio, *95% CI* 95% confidence interval, *Ref.* reference group

On the other hand, factors associated negatively with chlorine dioxide consumption as a treatment were strongly considering COVID-19 to be a dangerous and deadly disease (aPR: 0.56; 95%CI: 0.33–0.96), thinking that chlorine dioxide is not effective (aPR: 0.22; 95%CI: 0.12–0.42), and not being informed of the efficacy of chlorine dioxide (aPR: 0.15; 95%CI: 0.07–0.32) (Table 2).

## Discussion

### Main findings

The prevalence of chlorine dioxide consumption to prevent or treat COVID-19 was less than 20%. The factors associated with dioxide consumption both as prevention and treatment were to consider COVID-19 a dangerous and deadly disease and the opinion of chlorine dioxide Also, we found individual variables that was associated only to chlorine dioxide consumption for prevention, like sociodemographic variables (sex and age) and health variables (source where you obtain information about COVID-19, comorbidities, and use medications and medicinal plants for prevention or treatment of COVID-19).

### Chlorine dioxide consumption

Chlorine dioxide is a reactive synthetic gas that has been used in the paper industry, decontamination of public buildings, and water purification [5]. Its high reactivity explains its properties to eliminate microorganisms, however, this also determines the potential adverse effects [5]. Despite this, chlorine dioxide has been promoted as a cure for diseases such as malaria, HIV and cancer, among others [13]; it has also been proposed to prevent or treat COVID-19 [14]. However, there is no evidence on the efficacy of chlorine dioxide for such purposes, but there is evidence on its harmful effects, so institutions such as the Pan American Health Organization do not recommend its use [15].

Despite this, the present study found an approximate prevalence of 8 and 16% of chlorine dioxide consumption as prevention and treatment of COVID-19, respectively. We have not found studies published in scientific journals that have evaluated the prevalence of chlorine dioxide consumption in other countries. Although a previous study indicated that chlorine dioxide consumption in Peru is high, based on the number of related media articles and blog posts, a prevalence has not been specified [16]. This assumption is in agreement with what we found, and it must be taken into account that the prevalence in our study could be underestimated, given that most of the population evaluated had characteristics in common, such as educational level, urban residence, etc., that make them less susceptible to the consumption of these substances, as has been previously reported [17].

In addition, the prevalence of chlorine dioxide consumption as a treatment is twice as high as its consumption for prevention. This could be explained by the fact that the diagnosis of COVID-19 can generate greater anxiety [18], and, therefore, desperation to act against the condition, leading to a greater susceptibility to consume this type of substance that is sold as a “miracle cure” for the disease [19]. Both as prevention or treatment of COVID-19, the consumption of this substance can have fatal consequences. On the one hand, those who use it for prevention may neglect adherence to truly effective preventive measures, such as social distancing or the use of masks. On the other hand, those who use it for treatment may tend to experience additional complications due to the delay in seeking health care. Taking into account that in both cases there may also be cases of intoxication, its consumption has implications not only for health but also for higher healthcare costs and a greater burden on health systems [20].

### Factors associated with chlorine dioxide consumption

It was found that male participants consumed chlorine dioxide more frequently to prevent COVID-19. However, studies that evaluated the consumption of other substances (drugs) found no significant differences between the sexes (*p* > 0.05) [21]. Studies in other countries even found an association between female sex and the use of other substances (drugs, traditional medicine, vitamin C) for COVID-19 prevention (*p* < 0.05) [22]. This suggests the existence of factors specific to chlorine dioxide implicated in its higher consumption among the male population, such as the fact that the promoters of this substance were of the same sex, which may generate empathy in their peers [23]. Additionally, in Peru, according to the National Institute of Statistics and Informatics, men work more than women [24], a fact that could have led them to seek multiple prevention alternatives due to their greater exposure to COVID-19.

Likewise, it was found that being older than 30 years was associated with lower consumption of chlorine dioxide as prevention of COVID-19. In contrast to this, previous studies found heterogeneous results regarding the direction between age and the use of unconventional medical practices [25]. However, for this study, the association found is possibly due to the fact that older people do not rely as much on chemical products and prefer more natural alternatives for this purpose [26]. Another factor that could explain our result is that the older population in Peru does not usually use social networks as an informative medium [27], and this was the main source where campaigns promoting the use of chlorine dioxide were promoted [6], so they may have had less exposure to news promoting its use. However, some studies suggest that fake news may have a greater reach in older populations [28]. For this reason, governments should warn against waves of misinformation from popular social networks and other media.

Among the family factors studied, it was reasonable to expect that family members of health science students would have a lower prevalence of the use of substances without scientific evidence, such as chlorine dioxide. However, it was found that having a health sciences student increased the prevalence of consuming this substance to prevent COVID-19. This could be explained by the fact that at the family level the transmission of health information is based on experience, values, and customs [29], given that processes and ideologies that seek to protect health and manage disease triggers are developed in the domestic sphere [30]. In addition, the inexperience of the future physicians may have contributed to the use of chlorine dioxide by the other members. On the other hand, although in the bivariate analysis the presence of a health professional in the home was associated with a lower frequency of chlorine dioxide use, this was not the case when adjusted for the other variables. This could be due to the abundance of popular and scientific information without adequate quality [31], as well as a deficit in the applicability of the judicious use of evidence in health professionals in general [32].

Furthermore, it is noteworthy that having medical information (and not health professionals in general) as the main source of information about COVID-19 was associated with a lower prevalence of using chlorine dioxide as a preventive, which could be explained by the quality of information that a physician could provide about the false claims of chlorine dioxide. Also, the lack of an adequate source of information led many people to believe the sensationalist explanations of the product [33]. However, in the case of those who took chlorine dioxide as a treatment measure, the source of information was not associated, which leads us to believe that the fear of worsening or death from COVID-19 infection was greater and sufficient to influence their consumption, despite having received adequate information.

An interesting finding is that having comorbidities was associated with higher consumption of chlorine dioxide as COVID-19 prevention. These patients have a higher risk of mortality from COVID-19 [34], in addition to the fact that measures to treat their diseases may lead them to expose themselves more and decrease their preventive practices against COVID-19 [35]. Feeling more susceptible, the concern may lead them to seek complementary measures [36] such as evidence-free solutions to prevent the disease.

However, in the face of a disease detected as a threat, perceived susceptibility does not act the same as perceived severity [37]. In our study, people who considered COVID-19 a dangerous and deadly disease consumed less chlorine dioxide for prevention and treatment. This is similar to a study where it was found that being aware of COVID-19 severity may predict greater adherence to recommended prevention practices [38]. Both perceived severity and susceptibility depend on knowledge about the condition [37], so again, it is important to make efforts to combat infodemia and improve knowledge in the population so that people can improve their health practices and be able to recognize the ineffectiveness of practices without scientific evidence.

Concerning the use of other substances, it was found that the use of medicines and medicinal plants to prevent COVID-19 was associated with higher consumption of chlorine dioxide as a preventive measure. The use of medicinal plants [39] and self-medication are common practices in Peru, especially during the pandemic [21]. However, the fact that people consume one of these measures does not mean that they trust in its efficacy [40] leading them to seek more than one preventive measure such as medicines and to rely on pseudoscientific beliefs such as the consumption of chlorine dioxide [36]. This practice may result in the population having a false sense of security and relaxing preventive measures against COVID-19 [41].

It is crucial to highlight the importance of educating the population, which with an adequate source of information can learn about the damage that chlorine dioxide can have and its lack of benefits for COVID-19. As observed in our study, the belief that chlorine dioxide is not effective is associated with a lower consumption as prevention and treatment of COVID-19. The consumption of chlorine dioxide and its associated factors should be evaluated in other countries that have been under similar social and political influences as Peru. Information should be regulated by the media following the recommendations of institutions such as the Pan American Health Organization, which does not recommend chlorine dioxide, and not pseudoscientists.

### Limitations and strengths

The present study has limitations that should be taken into account. First, the non-probabilistic sampling used makes it difficult to extrapolate the prevalences of chlorine dioxide consumption found, since, in the sample studied, the high percentage of women would decrease them, while the high percentage of young people would increase them. Secondly, the survey was distributed virtually and to acquaintances of the authors and collaborators of the study, a fact that could have led to the characteristics found in the sample studied (high percentage of young people, with higher education and from the urban sector). Thirdly, the sample of patients with a history of COVID-19 was considerably smaller than those without COVID-19, making the estimates for the first group more imprecise. Fourthly, this study was a secondary analysis of previously collected data where excluded pregnant, for this reason, we can’t extrapolate our results to this specific population. However, the inclusion of this population could underestimate our general prevalence, because the women are more cautious in their decision about what things they can ingest.

Despite this, this study evaluated a total of 3610 individuals, including respondents with and without a history of COVID-19, from the 24 departments of Peru, and to our knowledge, this is the first study that evaluates the factors associated with chlorine dioxide consumption in the general Peruvian population, revealing a real public health problem.

## Conclusions

The prevalence of chlorine dioxide consumption among the Peruvian sample evaluated to prevent or treat COVID-19 was 8 and 16%, respectively. Considering COVID-19 dangerous and deadly, and considering that chlorine dioxide is not effective or not being informed of its efficacy were associated with lower consumption, both to prevent and treat COVID-19. Therefore, it is suggested to continue educating the population to avoid the consumption of this type of substance and the implementation of policies to regulate the information propagated by the media, to mitigate false news and promote evidence-based recommendations.

## Supplementary Information

**Additional file 1.**

**Additional file 2.**

## Data Availability

All data generated or analysed during this study are included in this published article and its supplementary information files.
