# Supplementary material for: Factors associated with the consumption of chlorine dioxide to prevent and treat COVID-19 in the Peruvian population: a cross-sectional study
Source: BMC Public Health. 2021 Nov 17;21:2109. doi: 10.1186/s12889-021-12191-9 (PMC8596383; doi:10.1186/s12889-021-12191-9)
Supplement: Supplementary file 1 — Additional file 1. [file 12889_2021_12191_MOESM1_ESM.docx]

## Supplementary material

**Supplementary Material 1. Questionnaire.**

****The original questionnaire is in Spanish***

**GENERAL DATA SECTION**

1. How old are you? ______________

2. What is your gender?

1. Woman
2. Man

3. What is your marital status?

1. Bachelor
2. Married or cohabiting
3. Divorced or Separated
4. Widower

4. What degree of instruction do you have?

1. Complete or incomplete primary
2. Incomplete secondary
3. Complete Secondary
4. Non-superior technician
5. Senior Technician
6. University Superior

5. What is your employment status?

1. Student
2. Unemployed
3. Independent work
4. Work in the public sector
5. Work in the private sector
6. Pensioner
7. Retiree
8. In perfect suspension

6. What kind of health insurance do you have?

1. You do not have any health insurance
2. SIS (MINSA)
3. ESSALUD
4. PRIVATE CLINIC
5. Armed Forces or PNP
6. OTHER

7. Where do you live? (DEPARTMENT)________________________________________

8. Do you currently live in a rural area?

1. Yes
2. No

9. What level of education does the head of the family have?

1. Complete or incomplete primary
2. Complete Secondary
3. Incomplete secondary
4. Non-superior technician
5. Complete Senior Technician
6. University Superior

10.What is the occupation of the head of the family?

1. University profession
2. Technical profession
3. Military or police
4. Trader or producer
5. Specialized employee
6. Non-specialized employee
7. Work in the informal sector

11.How do you consider your home to be?

1. Housing with the best sanitary conditions in luxury environments
2. Housing with the best sanitary conditions in low luxury environments
3. Housing with good sanitary conditions in comfortable environments
4. Housing with "deficiencies in some sanitary conditions"
5. Housing with "markedly inadequate" sanitary conditions

12. In your opinion: What is the family budget like?

1. Enough to solve family needs comfortably.
2. Just to solve the primary family needs.
3. Solves basic family needs with difficulty
4. It does not solve basic family needs

13. What is your family's main source of income?

1. Monthly salary
2. Weekly wage, per day, or fees
3. Professional earnings or fees
4. Inherited or acquired fortune
5. External financial support

14.Do you have a family member working in a hospital within your home?

1. Yes, a family health professional
2. Yes, I am a health professional
3. No, no one in my family is a health professional
4. No, but I have a family member who is a student in health sciences
5. No, but I am a student in health sciences

15.What source of information do you use the most, to be aware of COVID 19?

1. Social networks (Facebook, YouTube, Instagram, Twitter, etc.)
2. information from friends, family or neighbors (including WhatsApp messages)
3. Television or radio information
4. Media (newspapers, magazines, etc.)
5. Medical information (doctors, scientific journals, etc.)

16. Do you have any of the following health conditions? (YOU CAN DIAL MORE THAN ONE)

- Diabetes mellitus
- High blood pressure (high blood pressure)
- Obesity (Body Mass Index >=30)
- Chronic Renal Failure
- Asthma, cystic fibrosis, or COPD
- Immunosuppression (weakened immune system including Cancer)
- Serious heart condition (heart failure or history of heart attack)
- NONE

17. Does anyone in your household have any of the health conditions listed in question *17* or is there someone over the age of 65?

1. Yes
2. No

18. Were any members of your household diagnosed with COVID-19? (NOT INCLUDED)

1. Yes
2. No

19. Were you diagnosed with COVID-19?

1. Yes
2. No

**SECTION OF PRACTICES AND PERSPECTIVES FOR THOSE WHO WERE NOT DIAGNOSED WITH COVID-19**

20. In the last 2 weeks How many times...

|  | **0** | **1** | **2** | **3** | **4** | **5** | **6** | **7** | **8** | **9** | **10** | **11** | **12** | **13** | **≥14** |
| --- | --- | --- | --- | --- | --- | --- | --- | --- | --- | --- | --- | --- | --- | --- | --- |
| ... have you used medications to prevent COVID-19? |  |  |  |  |  |  |  |  |  |  |  |  |  |  |  |
| ... have you used medicinal plants in order to prevent COVID-19? |  |  |  |  |  |  |  |  |  |  |  |  |  |  |  |
| ... Have you used CHLORINE DIOXIDE in order to prevent COVID-19? |  |  |  |  |  |  |  |  |  |  |  |  |  |  |  |

21. What medicines do you take exclusively to prevent COVID-19? (YOU CAN DIAL MORE THAN ONE)

- None
- Ivermectin for human use
- Ivermectin for veterinary use
- Hydroxychloroquine
- Azithromycin or Clarithromycin
- Corticosteroids (dexamethasone, prednisone, etc.)
- Paracetamol
- NSAIDs (ibuprofen, naproxen, or diclofenac)
- Heparin or Warfarin (or another anticoagulant)
- OTHER

22. What medicinal plants do you use exclusively to prevent COVID 19? (YOU CAN DIAL MORE THAN ONE)

- None
- Eucalyptus
- Matico
- Mallow
- Plantain
- Quinine
- Lemon verbena
- Cypress
- Soursop Leaves
- Lemon Leaves
- Kion
- Bands
- OTHER (garlic, etc. )
  23. As a preventive measure, what do you think about the use:

|  | It's effective, it does good | It's effective, it does little harm | Not effective, but it doesn't hurt | It is not effective and does harm | I am not informed of that issue |
| --- | --- | --- | --- | --- | --- |
| Ivermectin |  |  |  |  |  |
| Other medicines |  |  |  |  |  |
| Chlorine Dioxide |  |  |  |  |  |
| Medicinal plants |  |  |  |  |  |

24. To what extent do you CONSIDER that taking medicines, plants, or other substances protect against getting sick from COVID-19?

1. A lot
2. Little
3. Nothing

25. To what extent do you CONSIDER COVID-19 to be a dangerous and deadly disease?

1. A lot
2. Little
3. Nothing

HERE ENDS THE SURVEY, THANK YOU VERY MUCH FOR YOUR TIME

************************************************************************************************************

**SECTION OF PRACTICES AND PERSPECTIVES FOR THOSE WHO WERE DIAGNOSED WITH COVID-19**

20. During your illness with COVID-19 How many times...

|  | **0** | **1** | **2** | **3** | **4** | **5** | **6** | **≥7** |
| --- | --- | --- | --- | --- | --- | --- | --- | --- |
| ... have you used medicines against COVID-19? |  |  |  |  |  |  |  |  |
| ... have you used medicinal plants against COVID-19? |  |  |  |  |  |  |  |  |
| ... have you used CHLORINE DIOXIDE against COVID-19? |  |  |  |  |  |  |  |  |

21. What medicines have you used against COVID-19? (YOU CAN DIAL MORE THAN ONE)

- None
- Ivermectin for human use
- Ivermectin for veterinary use
- Hydroxychloroquine
- Azithromycin or Clarithromycin
- Chlorine dioxide
- Corticosteroids (dexamethasone, prednisone, etc.)
- Paracetamol
- NSAIDs (ibuprofen, naproxen, or diclofenac)
- Heparin or Warfarin (or another anticoagulant)
- Other...

22. What medicinal plants do you use or have you used against COVID-19? (YOU CAN DIAL MORE THAN ONE)

- None
- Eucalyptus
- Matico
- Mallow
- Plantain
- Quinine
- Lemon verbena
- Soursop Leaves
- Lemon Leaves
- Kion
- Cypress
- Bands
- Garlic
- OTHER

23. As a treatment measure, what do you think about the use:

|  | It's effective, it does good | It's effective, it does little harm | Not effective, but it doesn't hurt | It is not effective and does harm | I am not informed of that issue |
| --- | --- | --- | --- | --- | --- |
| Ivermectin |  |  |  |  |  |
| Other medicines |  |  |  |  |  |
| Chlorine Dioxide |  |  |  |  |  |
| Medicinal plants |  |  |  |  |  |

24. To what extent do you CONSIDER that taking medicines, plants, or other substances protect against getting sick from COVID-19?

1. A lot
2. Little
3. Nothing

25. To what extent do you CONSIDER COVID-19 to be a dangerous and deadly disease?

1. A lot
2. Little
3. Nothing

HERE ENDS THE SURVEY, THANK YOU VERY MUCH FOR YOUR TIME

************************************************************************************************************

**Supplementary material 2. Participant selection flowchart.**

Pregnant at survey

N=20

Patients in the initial database

N=3630 (3231 without COVID-19 and 399 with COVID-19)

Total respondents

N=3610 (3213 without COVID-19 and 397 with COVID-19)
